# Fibromodulin-Deficiency Alters Temporospatial Expression Patterns of Transforming Growth Factor-β Ligands and Receptors during Adult Mouse Skin Wound Healing

**DOI:** 10.1371/journal.pone.0090817

**Published:** 2014-03-06

**Authors:** Zhong Zheng, Kevin S. Lee, Xinli Zhang, Calvin Nguyen, Chingyun Hsu, Joyce Z. Wang, Todd Matthew Rackohn, Dwarak Reddy Enjamuri, Maxwell Murphy, Kang Ting, Chia Soo

**Affiliations:** 1 Dental and Craniofacial Research Institute and Section of Orthodontics, School of Dentistry, University of California Los Angeles, Los Angeles, California, United States of America; 2 Department of Emergency Medicine, State University of New York Downstate/Kings Country Hospital Center, New York, New York, United States of America; 3 Department of Psychobiology, University of California Los Angeles, Los Angeles, California, United States of America; 4 UCLA and Orthopaedic Hospital Department of Orthopaedic Surgery and the Orthopaedic Hospital Research Center, University of California Los Angeles, Los Angeles, California, United States of America; 5 Division of Plastic and Reconstructive Surgery, Department of Surgery, School of Medicine, University of California Los Angeles, Los Angeles, California, United States of America; The University of Hong Kong, Hong Kong

## Abstract

Fibromodulin (FMOD) is a small leucine-rich proteoglycan required for scarless fetal cutaneous wound repair. Interestingly, increased FMOD levels have been correlated with decreased transforming growth factor (TGF)-β1 expression in multiple fetal and adult rodent models. Our previous studies demonstrated that FMOD-deficiency in adult animals results in delayed wound closure and increased scar size accompanied by loose package collagen fiber networks with increased fibril diameter. In addition, we found that FMOD modulates *in vitro* expression and activities of TGF-β ligands in an *isoform-specific* manner. In this study, temporospatial expression profiles of TGF-β ligands and receptors in FMOD-null and wild-type (WT) mice were compared by immunohistochemical staining and quantitative reverse transcriptase-polymerase chain reaction using a full-thickness, primary intention wound closure model. During the inflammatory stage, elevated inflammatory infiltration accompanied by increased type I TGF-β receptor levels in individual inflammatory cells was observed in FMOD-null wounds. This increased inflammation was correlated with accelerated epithelial migration during the proliferative stage. On the other hand, significantly more robust expression of TGF-β3 and TGF-β receptors in FMOD-null wounds during the proliferative stage was associated with delayed dermal cell migration and proliferation, which led to postponed granulation tissue formation and wound closure and increased scar size. Compared with WT controls, expression of TGF-β ligands and receptors by FMOD-null dermal cells was markedly reduced during the remodeling stage, which may have contributed to the declined collagen synthesis capability and unordinary collagen architecture. Taken together, this study demonstrates that a single missing gene, *FMOD*, leads to conspicuous alternations in TGF-β ligand and receptor expression at all stages of wound repair in various cell types. Therefore, FMOD critically coordinates temporospatial distribution of TGF-β ligands and receptors *in vivo*, suggesting that FMOD modulates TGF-β bioactivity in a complex way beyond simple physical binding to promote proper wound healing.

## Introduction

Wound healing is a highly ordered and well-coordinated process involving inflammation, proliferation, and remodeling orchestrated by a myriad of cytokines and growth factors [1]–[3]. Transforming growth factor (TGF)-β, which is like a cytokine owing to its small molecular weight and selective effect on multiple inflammatory processes, is one of the most crucial modulators in wound healing [1]–[6]. TGF-β is produced by several cell types that are present in wound site including activated macrophages, neutrophils, platelets, fibroblasts, and keratinocytes [3], [5], [7]. Three mammalian TGF-β isoforms (TGF-β1, β2, and β3), which share 64–86% amino acid sequence homology, are all essential for wound healing regulation [5], [8], [9]. Interestingly, although all three TGF-β isoforms exert their effects by binding to transmembrane type II receptor (TβRII), which activates type I receptor (TβRI) to initiate signal transduction [10] they exhibit different and even opposite functions. For example, compared with TGF-β1 and β2, TGF-β3 presents more potent inhibition on DNA synthesis in keratinocytes [11]. Moreover, TGF-β1 significantly promotes migration of dermal fibroblasts, while TGF-β3 exerts remarkable anti-migratory effects on these cells [12]–[14]. In addition, TGF-β1 and β2 are known to promote fibroplasia, while TGF-β3 may or may not reduce scar [15]–[20]. It is worthy of note that TGF-βs can bind to a variety of extracellular matrix (ECM) proteins and regulate their expression and activities [21]–[26], and these ECM molecules in turn can modulate TGF-β expression and activities [21], [27]–[29]. For instance, application of decorin, a small leucine-rich proteoglycan (SLRP) family member, for prevention of TGF-β-mediated fibrosis in various tissues is currently being attempted due to the high-affinity interactions between decorin and TGF-β isoforms [27]–[31].

To date, 18 SLRP family members have been discovered, which are grouped into five classes based mainly on evolutionary conservation, homology, and chromosomal organization [32]. Regardless of the classification, SLRPs share common functionality including interaction with diverse molecules such as TGF-βs, collagens, and other ECM molecules, and thus have wide-ranging functions from regulation of collagen matrix architecture and mechanical properties to control of cellular proliferation and differentiation [32]–[34]. Notably, fibromodulin (FMOD), a class II keratin sulphate SLRP, is essential for maintenance of endogenous stem cell niches [35]. In fact, FMOD-deficiency leads to osteogenesis of tendon stem/progenitor cells [35], resulting in a structurally and mechanically abnormal tendon phenotype [36], [37]. Moreover, recent studies demonstrated that FMOD can directly reprogram somatic cells to a minimally proliferative, multipotent progenitor state [38], indicating that FMOD regulates intracellular signaling cascade and determines cell fate in addition to carrying out ECM structural functions [39].

In terms of skin wound repair, our previous studies have demonstrated that high expression of FMOD in fetal skin decreases during the transition from scarless fetal-type repair to adult-type repair with scarring [40], [41]. Likewise, adult animals with FMOD-deficiency display abnormal wound healing: they exhibit delayed dermal wound closure and increased scar formation with reduced angiogenesis and unordinary collagen architecture [14], [42]. Importantly, when we used FMOD to rescue rat fetal wounds at day 19 of gestation (E19) from scar formation, TGF-β1 expression was significantly decreased [40]. We have also found that FMOD modulates expression and activities of TGF-βs in an *isoform-specific* manner [14], [40], [41]. For example, FMOD potentiates TGF-β1-induced cell migration and reverses TGF-β3 inhibition of migration, governing timely epithelialization and granulation tissue formation. Thus, the relationship among FMOD expression, TGF-β activity, and scarring in both fetal and adult wound repair suggests that FMOD may exert anti-scarring effects by orchestrating TGF-β expression and function.

In this study, we investigated the temporospatial distribution of TGF-β ligands and receptors in both adult FMOD-null and wild-type (WT) mouse wounds to further elucidate how FMOD coordinates TGF-β bioactivity to promote proper cutaneous wound healing.

## Materials and Methods

### Ethics statement

All animal surgery procedures were performed under institutionally approved protocols provided by the Chancellor's Animal Research Committee at UCLA (protocol number: 2000–058).

### Primary intention wound closure model

Three and one-half month old male 129/sv WT and FMOD-null mice [36] were anesthetized, and the dorsal skin was sterilely prepared [14]. Four full-thickness, 10 mm×3 mm skin ellipses with the underlying *panniculus carnosus* muscle were excised on each mouse. Each open wound edge was injected with 25 µl phosphate-buffered saline or 0.4 mg/ml recombinant FMOD solution (25 µl×2 edges  = 50 µl total per wound). Wounds were then closed primarily with 4–0 Nylon using two simple interrupted sutures consistently placed at one-third intervals in each 10 mm length wound. All wounds were separated by at least 2 cm to minimize adjacent wound effects [14]. Sutures were removed at day 7 post-injury and wounds were harvested at 0.5, 1, 2, 3, 5, 7 and 14 days after injury. Unwounded skin samples from identical locations in three animals were collected as controls. Tissue for RNA isolation (entire wound +1 mm edge) was immediately frozen in liquid nitrogen and stored at −80°C until RNA extraction [14]. Wound tissue for histology (entire wound +10 mm edge) was bisected centrally between the two 4–0 Nylon sutures, perpendicular to the long axis of each 10 mm length wound, and placed in formalin [14].

### Histology and immunohistochemistry

After fixation, skin samples (from 36 wounds of 16 animals per time point) were dehydrated, paraffin-embedded, and cut into 5-µm sections for hematoxylin and eosin (H&E) and immunohistochemical (IHC) staining. To ensure consistent sampling from the center rather than the periphery of the wound, specimens were sectioned starting from the areas of previous wound bisection. Immunohistochemistry staining was performed as previously described [43]. Relative inflammatory infiltration in 8 animals per genotype (2 randomly chosen wound edge fields and 2 randomly chosen wound bed fields per animal) was semi-quantitatively evaluated by three blinded reviewers using the following criteria:

Control: unwounded WT skin tissue (≤2 inflammatory cells per field based on 32 randomly selected fields from 16 unwounded WT animals)

Score 0: <2 times the unwounded skin tissue

Score 1: 2–5 times the unwounded skin tissue

Score 2: 5–10 times the unwounded skin tissue

Score 3: 10–15 times the unwounded skin tissue

Score 4: >15 times the unwounded skin tissue.

All antibodies used in this study were obtained from Santa Cruz Biotech. (Santa Cruz, CA) except for the antibody against lumican that was purchased from Abcam Inc. (Cambridge, MA) (**Table 1**). Computerized immunolocalization intensity analysis was performed using the commercial software Image-Pro® Plus 6.0 (Media Cybernetics Inc., Rockville, MD). Relative dermal protein expression was quantified by the mean optical density of staining signal × percent area positively stained ×100 [14], [44].

**Table 1:** Description of Primary Antibodies Used for Indirect Immunostaining.

Antibody	Initial Concentration	Origin	Epitope recognition	Final concentration (diluted in PBS)
TGF-β1 (V)	200 µg/ml	Rabbit polyclonal IgG	Extreme carboxy terminus of TGF-β1 (region of mature peptide)	0.002 g/L (1:100)
TGF-β2 (V)	200 µg/ml	Rabbit polyclonal IgG	Extreme carboxy terminus of TGF-β2 (region of mature peptide)	0.002 g/L (1:100)
TGF-β3 (V)	200 µg/ml	Rabbit polyclonal IgG	Extreme carboxy terminus of TGF-β3 (region of mature peptide)	0.002 g/L (1:100)
TβRI (V-22)	200 µg/ml	Rabbit polyclonal IgG	Carboxy terminus of TβRI (region of mature peptide)	0.002 g/L (1:100)
TβRII (C-16)	200 µg/ml	Rabbit polyclonal IgG	Carboxy terminus of TβRII (region of mature peptide)	0.002 g/L (1:100)
TβRIII (C-20)	200 µg/ml	Goat polyclonal IgG	Carboxy terminus of TβRIII (region of mature peptide)	0.002 g/L (1:100)
Lumican(L-20)	1000 µg/ml	Rabbit monoclonal IgG	Internal region of lumican	0.02 g/L (1:50)

### Quantitative reverse transcriptase-polymerase chain reaction (qRT-PCR)

Total RNA was extracted using TRIzol® Reagent (Invitrogen, Carlsbad, CA) and treated with RNase-Free DNase Set (Qiagen, Valencia, CA) to remove chromosomal DNA contamination. 1 µg total RNA was reverse transcribed into cDNA in a 20-µl reaction mixture with 50 pmol of oligo(dT)_20_ primer and 1 µl (200 U) of SuperScript^TM^ III reverse transcriptase (Invitrogen). Expression of mRNA was measured by qRT-PCR using TaqMan® Gene Expression Assays (**Table 2**) on a 7500 Fast Real-time PCR System (Applied Biosystems, Foster City, CA) [14]. Concomitant glyceraldehyde-3-phosphate dehydrogenase (GAPDH) was also performed in separate tubes for each RT reaction with TaqMan® Rodent GAPDH control reagents (Applied Biosystems). For each gene, at least three separate sets of qRT-PCR analysis were performed using different complementary DNA templates.

**Table 2:** TaqMan® Gene Expression Assay.

Gene	Genbank no.	TaqMan® Gene Expression Assay no.*
TGF-β1	NM_011577.1	Mm00441724_m1
TGF-β2	NM_009367.1	Mm00436952_m1
TGF-β3	NM_009368.1	Mm00436960_m1
TβRI	NM_009370.2	Mm00436971_m1
TβRII	NM_009371.2	Mm00466494_s1
TβRIII	NM_011578.2	Mm00803538_m1

* Commercial TaqMan® Gene Expression Assays were purchased from Applied Biosystems, Foster City, CA, USA.

### Scratch wound assay

Primary dermal fibroblasts from WT and adult FMOD-null mice were derived and maintained following the published procedure [45]. A scratch wound assay was performed to mimic the scarring process *in vitro* [46]. Briefly, passage 3 dermal fibroblasts were grown in 6-well tissue culture plates until confluence. After 12 h serum starvation, the cell monolayer was scraped to generate a single 1-mm wide gap. The wounded monolayer was washed 3 times with phosphate-buffered saline (PBS) to remove dead cells prior to 24 h incubation in DMEM medium (Invitrogen). 100 pM TGF-β1 or -β3 (Sigma-Aldrich, St. Louis, MO) in PBS was used to regulate fibroblast migration [14], while 10 µM TβRI-specific inhibitor SB-431542 (Sigma-Aldrich) [47] was used to attenuate TβRI-mediated TGF-β bioactivities. Cell migration was documented by photographs taken immediately after scraping, as well as 24 h later. Migration was quantified by measuring the average wound gap between the wound edges before and after the treatment using the commercial software Image-Pro® Plus 6.0, and calculated as: Cell migration (%)  =  (Gap_0h_-Gap_24h_)/Gap_0h_ ×100%.

### Statistical analysis

The results are graphically depicted as the mean ± the standard error of mean. Statistical significance was computed using ANOVA (13.0 for Windows, SPSS, Chicago, IL). Independent-sample *t*-test was used to compare results of two groups. Individual comparisons between two groups were determined using the Mann-Whitney test for non-parametric data. *P* value <0.05 was considered statistically significant.

## Results

### FMOD-null mice exhibit increased inflammation

**Figure 1:**
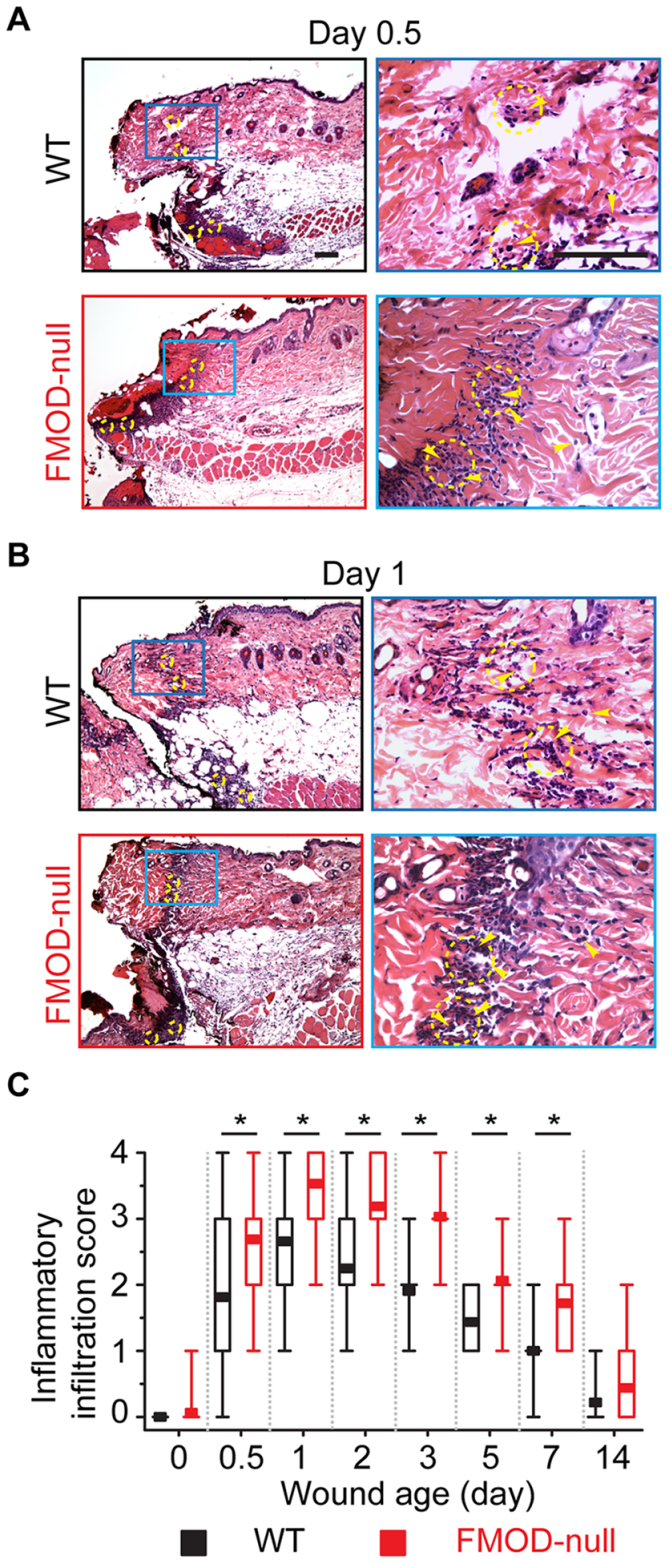
Hematoxylin and eosin (H&E) staining of wounded WT and FMOD-null adult mice skin. (**A**) At day 0.5 post-injury, minimal (score: 1) inflammatory infiltrate was present at the wound edge of WT mice (*upper right*), while significant (score: 3) inflammatory infiltrate was detected at the wound base. On the other hand, moderate (score: 2) and significant inflammatory infiltrates were observed at the wound edge (*lower right*) and base of FMOD-null mice, respectively. (**B**) At day 1 post-injury, moderate and significant inflammatory infiltrates were seen at the wound edge (*upper right*) and base of WT mice, respectively. Meanwhile, high (score: 4) inflammatory infiltrate was observed at both the wound edge (*lower-right*) and base of FMOD-null mice. (**C**) Relative inflammatory infiltration (median, 25–75% quartile, min, max) in 8 animals per genotype (2 randomly chosen wound edge fields and 2 randomly chosen wound bed fields per animal; N = 32) was semi-quantitatively evaluated by three blinded reviewers. Yellow arrowheads: representative inflammatory cells (not all inflammatory cells are indicated); yellow circles: randomly chosen fields for inflammatory infiltration evaluation. Bar =  100 µm. *, significant difference determined by the Mann-Whitney test.

Wound inflammatory infiltration was scored from 1 (minimal) to 4 (high) by three independent pathologists using the criteria described in **Materials and Methods** section. At day 0.5 post-injury, WT wounds showed minimal inflammatory cell numbers (score: 1) within the fibrin clot at the wound edge and significant inflammatory infiltrate (score: 3) at the wound base upon H&E staining (**Figure 1A**). At the same locations, FMOD-null wounds showed moderate inflammatory cell numbers (score: 2) at the wound edge and, like WT wounds, significant inflammatory infiltrate (score: 3) at the wound base (**Figure 1A**). For both genotypes, neutrophils comprised the major inflammatory cell type present at day 0.5 post-injury, followed by monocytes (about 10%) and lymphocytes (about 5%). At day 1 post-injury, WT wounds exhibited mildly to moderately elevated inflammatory infiltrate at both the wound edge and wound base (score: 2 to 3) (**Figure 1B**), with a higher lymphocyte proportion at the wound periphery (about 15%). In contrast, highly increased inflammatory infiltrate (score: 4) was observed at both the wound edge and wound base of FMOD-null mice at day 1 post-injury (**Figure 1B**). Macrophages were noted 1 day after injury at the wound base of FMOD-null mice, and not until 2 days after injury in WT mice. Overall, FMOD-null mice demonstrated higher inflammatory infiltration than WT animals before wound closure (**Figure 1C**), and inflammatory cell density was significantly reduced with macrophages as the dominant immune cell type (above 50%) in both genotypes after would closure.

### TGF-β1 protein expression is elevated in migrating epidermis and granulation tissue of FMOD-null wounds

**Figure 2:**
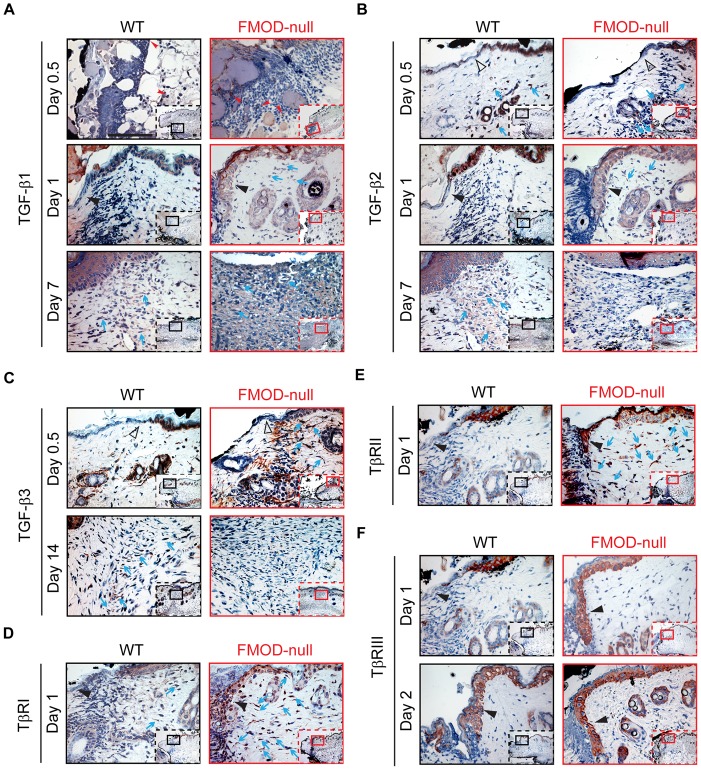
Immunohistochemical (IHC) staining of wounded WT and FMOD-null adult mice skin. (**A**) TGF-β1, (**B**) TGF-β2, (**C**) TGF-β3, (**D**) TβRI, (**E**) TβRII, and (**F**) TβRIII. Inserts show low magnification view. Red arrowheads: inflammatory cells; open black triangles: epidermis at wound edge; solid black triangles: migrating epidermal tongues; blue arrows: dermal fibroblasts. Bar  = 100 µm.

**Figure 3:**
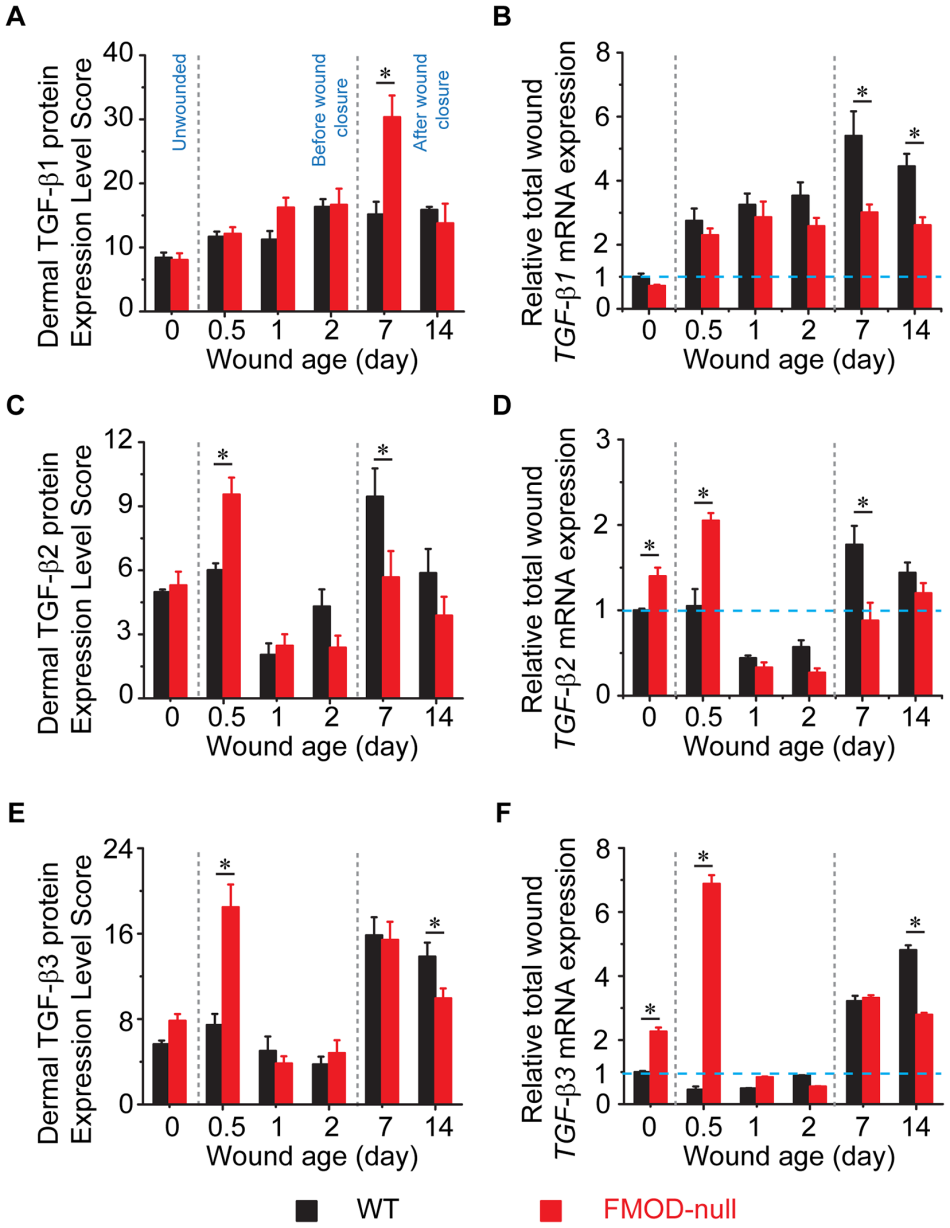
Quantification of dermal protein expression (A, C, E; N = 9) and total wound RNA (B, D, F; N = 4) expression of TGF-β ligands. (**A, B**) TGF-β1, (**C, D**) TGF-β2, and (**E, F**) TGF-β3. RNA expression is normalized to unwounded WT skin (blue dotted line). *, *P*<0.05.

**Figure 4:**
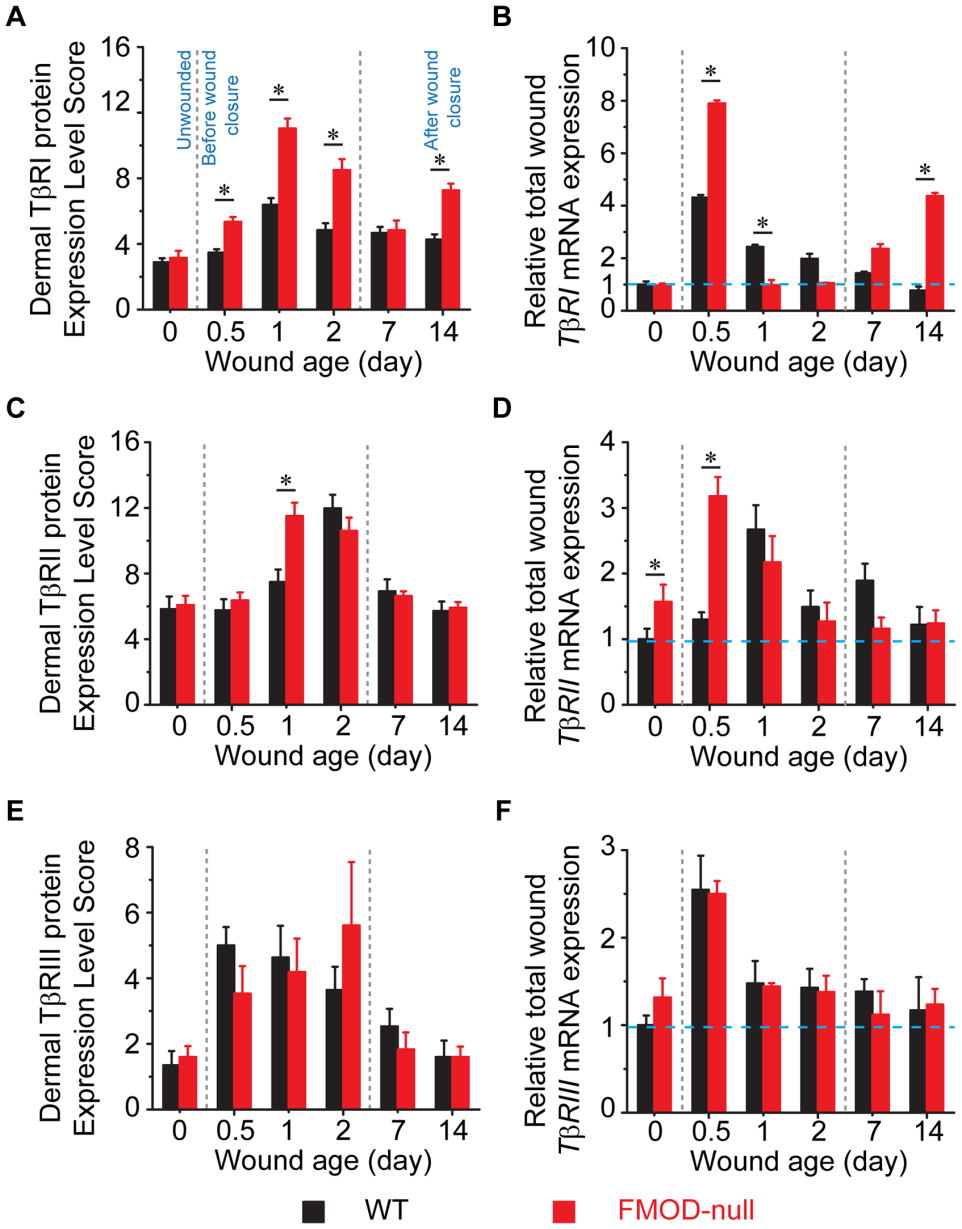
Quantification of dermal protein expression (A, C, E; N = 9) and total wound RNA (B, D, F; N = 4) expression of TGF-β receptors. (**A, B**) TβRI, (**C, D**) TβRII, and (**E, F**) TβRIII. RNA expression is normalized to unwounded WT skin (blue dotted line). *, *P*<0.05.

To determine how FMOD-deficiency affects wound healing, expression of TGF-β ligands and receptors was documented in adult FMOD-null mice and compared with that in WT controls. Cellular and ECM TGF-β ligand and receptor immunostaining patterns are shown in **Figure 2** (which provides information on global TGF-β ligand and receptor staining and relative cell density), while the dermal protein expression and relative total wound mRNA transcription levels are summarized in **Figure 3** (for TGF-β ligands) and **Figure 4** (for TGF-β receptors). In addition, specific staining intensities for epidermal, dermal, inflammatory, and hair follicle cell types are summarized in **Tables 3** and **4** (which provide information on individual cell TGF-β ligand and receptor staining intensities, respectively).

**Table 3:** Relative Immunostaining Intensity of TGF-β Ligands in Wounded WT and FMOD-null Adult Mice*.

After injury	Unwounded		0.5 day		1 day		2 days		7 days		14 days	
TGF-β1	WT*	FN*	WT	FN	WT	FN	WT	FN	WT	FN	WT	FN
Epidermis												
Migrating epi.	N/A*	N/A	N/A	N/A	−*	+++*	+++	+++	N/A	N/A	N/A	N/A
Outer layer	+++	+++	+++	+++	+++	+++	+++	+++	+++	+++	+++	+++
Basal layer	+++	+++	+++	+++	+++	+++	+++	+++	+++	+++	+++	+++
Dermis												
ECM	−	−	−	−	−	−	−	−	+*	+++	+	+
Fibroblasts	−	−			−	++*	++	++	+++	+++	+++	+++
Inflammatory cells†	−	−	++	++	++	++	+++	+++	+++	+++	+++	+++
Hair follicles‡	+++	+++	+++	+++	+++	+++	+++	+++	+++	+++	+++	+++

After injury	Unwounded		0.5 day		1 day		2 days		7 days		14 days	
TGF-β2	WT	FN	WT	FN	WT	FN	WT	FN	WT	FN	WT	FN
Epidermis												
Migrating epi.	N/A	N/A	N/A	N/A	−	+++	+++	+++	N/A	N/A	N/A	N/A
Outer layer	+++	+++	−	−	+++	+++	+++	+++	+++	+++	+++	+++
Basal layer	+++	+++	−	−	+++	+++	+++	+++	+++	+++	+++	+++
Dermis												
ECM	−	−	−	−	−	−	−	−	++	+	+	+
Fibroblasts	−	−	+++	+++	−	+++	+	+++	+++	−	−	−
Inflammatory cells	−	−	+++	+++	+++	+++	++	++	−	−	−	−
Hair follicles	+++	+++	+++	+++	+++	+++	+++	+++	+++	+++	+++	+++

After injury	Unwounded		0.5 day		1 day		2 days		7 days		14 days	
TGF-β3	WT	FN	WT	FN	WT	FN	WT	FN	WT	FN	WT	FN
Epidermis												
Migrating epi.	N/A	N/A	N/A	N/A	−	+++	+++	+++	N/A	N/A	N/A	N/A
Outer layer	+++	+++	−	−	+++	+++	+++	+++	++	++	++	++
Basal layer	+++	+++	−	−	+++	+++	+++	+++	+++	+++	+++	+++
Dermis												
ECM	−	−	−	+++	+	++	−	−	+	+	+	−
Fibroblasts	−	−	−	+++	+	++	+	+	++	++	+++	−
Inflammatory cells	−	−	−	−	++	+	−	++	−	−	−	−
Hair follicles	+++	+++	−	−	+++	+++	+++	+++	+++	+++	+++	+++

* WT, wild-type; FN, FMOD-null; N/A, not applicable; −, negligible staining (<5%); +, minimal staining (5%–25%); ++, moderate staining (25%–50%); +++, strong staining (>50%).

† In general, unwounded control skin contained very few inflammatory cells. The chart reflects the intensity of intracellular TGF-β staining and not the actually number of inflammatory cells present.

‡ Due to no hair follicle regeneration was detected in the adult skin wounds in this study, the chart reflects the influence of the wounds on adjacent hair follicles.

**Table 4:** Relative Immunostaining Intensity of TGF-β Receptors in Wounded WT and FMOD-null Adult Mice.

After injury	Unwounded		0.5 day		1 day		2 days		7 days		14 days	
TβRI	WT*	FN*	WT	FN	WT	FN	WT	FN	WT	FN	WT	FN
Epidermis												
Migrating epi.	N/A*	N/A	−*	−	−	+++*	+++	+++	N/A	N/A	N/A	N/A
Outer layer	+++	+++	−	−	+++	+++	+++	+++	+++	+++	+++	+++
Basal layer	+++	+++	−	−	+++	+++	+++	+++	+++	+++	+++	+++
Dermis												
ECM	−	−	−	−	−	−	−	−	−	−	−	−
Fibroblasts	+++	+++	−	++*	+++	+++	+++	+++	+++	+++	+++	+++
Inflammatory cells†	−	−	−	++	+++	+++	+++	+++	−	−	−	−
Hair follicles‡	+++	+++	−	−	+*	+	+++	+++	+++	+++	+++	+++

After injury	Unwounded		0.5 day		1 day		2 days		7 days		14 days	
TβRII	WT	FN	WT	FN	WT	FN	WT	FN	WT	FN	WT	FN
Epidermis												
Migrating epi.	N/A	N/A	−	+++	−	+++	+++	+++	N/A	N/A	N/A	N/A
Outer layer	+++	+++	−	+++	+++	+++	+++	+++	+++	+++	+++	+++
Basal layer	+++	+++	−	+++	+++	+++	+++	+++	+++	+++	+++	+++
Dermis												
ECM	−	−	−	−	−	−	−	−	−	−	−	−
Fibroblasts	+	+	−	+++	−	+++	+++	+++	+++	+++	+	+
Inflammatory cells	−	−	−	−	+++	+++	+++	+++	++	++	−	−
Hair follicles	+++	+++	−	+++	−	+++	+++	+++	+++	+++	+++	+++

After injury	Unwounded		0.5 day		1 day		2 days		7 days		14 days	
TβRIII	WT	FN	WT	FN	WT	FN	WT	FN	WT	FN	WT	FN
Epidermis												
Migrating epi.	N/A	N/A	−	−	−	+++	+++	+++	N/A	N/A	N/A	N/A
Outer layer	+++	+++	+++	+++	+++	+++	+++	+++	++	+++	+++	++
Basal layer	+++	+++	+++	+++	+++	+++	+++	+++	+++	+++	+++	+++
Dermis												
ECM	−	−	−	−	−	−	−	−	−	−	−	−
Fibroblasts	−	−	−	−	−	−	−	−	−	−	−	−
Inflammatory cells	−	−	−	−	−	−	−	−	−	−	−	−
Hair follicles	+++	+++	−	−	+	+++	++	+++	++	+++	+++	+++

* WT, wild-type; FN, FMOD-null; N/A, not applicable; −, negligible staining (<5%); +, minimal staining (5%–25%); ++, moderate staining (25%–50%); +++, strong staining (>50%).

† In general, unwounded control skin contained very few inflammatory cells. The chart reflects the intensity of intracellular TGF-β receptor staining and not the actually number of inflammatory cells present.

‡ Due to no hair follicle regeneration was detected in the adult skin wounds in this study, the chart reflects the influence of the wounds on adjacent hair follicles.

During the wound healing process, although the relative inflammatory infiltrate (primarily comprised of neutrophils, monocytes, and macrophages) was greater in FMOD-null wounds compared with WT wounds (**Figure 1**), the TGF-β1 staining intensity for the individual inflammatory cells was relatively similar between WT and FMOD-null wounds (**Figure 2A** and **Table 3**). Strong (+++) TGF-β1 staining was observed at the epithelial wound edge in both WT and FMOD-null genotypes (**Table 3**). TGF-β1 staining was detected at the migrating epidermis at day 1 post-injury in FMOD-null mice, while TGF-β1 staining was hardly observed at the migrating epidermis in WT mice at the same wound healing stage (**Figure 2A** and **Table 3**). With respect to fibroblasts, both WT and FMOD-null mice exhibited negligible (−) TGF-β1 staining prior to injury, but TGF-β1 signals were increased to moderate (++) levels in about 10% of FMOD-null fibroblasts at day 1 post-injury and in WT fibroblasts at day 2 (**Table 3** and **Figure 3A**). These observations correlated with the qRT-PCR data demonstrating similar *TGF-β1* expression levels in WT and FMOD-null mice before injury and before wound closure (**Figure 3B**). However, after wound closure (day 3 for WT wounds and day 5 for FMOD-null wounds [14]), TGF-β1 staining intensity in WT and FMOD-null fibroblasts increased to strong levels at days 7 and 14 (**Figure 2A** and **Table 3**). At day 7 post-injury, ECM of WT granulation tissue showed only minimal TGF-β1 signals, while ECM of FMOD-null granulation tissue showed strong TGF-β1 staining that contributed to significantly higher total dermal TGF-β1 protein expression levels in FMOD-null wounds (**Table 3**, **Figures 2A** and **3A**). Peak TGF-β1 ECM staining at day 7 coincided with peak fibroblast density rather than peak inflammatory cell density in FMOD-null wounds – suggesting that the source of elevated ECM TGF-β1 in FMOD-null wounds is likely fibroblasts rather than inflammatory cells. Interestingly, total *TGF-β1* mRNA expression when normalized to *GAPDH* was significantly higher in WT wounds than in FMOD-null wounds during the entire 14-day experimental period (**Figure 3B**), while total dermal TGF-β1 protein expression levels were significantly higher in FMOD-null wounds at day 7 post-injury (**Figure 3A**). The reason for this apparent discrepancy between TGF-β1 mRNA and protein expression profiles at day 7 may be that qRT-PCR results are reflective of gene expression per cell, and thus do not take into account the increased cell density in day 7 FMOD-null granulation tissue (**Figure 2A** and *ref.* [14]). In contrast, IHC results are reflective of total TGF-β1 protein expression quantity in all the cells present within the granulation tissues. Cell density was significantly higher in FMOD-null wounds at day 7 post-injury, but a single WT cell may have had a relatively higher *TGF-β1* mRNA transcriptional activity than a single FMOD-null cell. Thus, the total amount of stained TGF-β1 protein was higher in FMOD-null wounds at day 7 post-injury. Overall, WT and FMOD-null wounds demonstrated similar TGF-β1 staining intensities for inflammatory cells, epidermal cells, hair follicles, and fibroblasts. However, epidermal TGF-β1 staining was significantly more pronounced in FMOD-null mice relative to WT mice at day 1 post-injury, while TGF-β1 staining in ECM of granulation tissue was more prominent in FMOD-null mice at day 7 post-injury.

### TGF-β2 expression in FMOD-null wounds is increased at early wound stage but decreased after wound closure

After injury, inflammatory cell TGF-β2 staining intensity was increased to strong levels at day 0.5 post-wounding, but reduced to negligible levels at days 7 and 14 in WT and FMOD-null mice (**Table 3**). Unwounded skin exhibited strong TGF-β2 staining in epidermis and hair follicles, but TGF-β2 levels in WT and FMOD-null epithelial wound edges dropped to negligible levels without a marked difference between the two genotypes at day 0.5 post-injury (**Table 3**). With respect to fibroblasts, WT displayed strong TGF-β2 staining intensity at day 0.5 post-injury that diminished by day 1 post-injury, followed by an upswing in TGF-β2 expression that peaked at day 7 (**Figure 2B** and **Table 3**). Meanwhile, FMOD-null fibroblasts presented strong TGF-β2 signals during day 0.5 to day 2 post-injury that disappeared after wound closure (**Figure 2B** and **Table 3**). After wound closure, TGF-β2 signals were moderate in WT granulation tissue ECM at day 7 that decreased to minimal levels by day 14 post-injury (**Figure 2B** and **Table 3**), while minimal TGF-β2 staining was observed in FMOD-null granulation tissue ECM at days 7 and 14 (**Figure 2B**, **Table 3**). In addition, total dermal TGF-β2 protein staining (**Figure 3C**) and *TGF-β2* mRNA transcriptional activity (**Figure 3D**) were significantly higher in FMOD-null wounds at day 0.5 post-injury relative to WT wounds. Unlike TGF-β1, both total dermal TGF-β2 staining (**Figure 3C**) and *TGF-β2* mRNA transcription (**Figure 3D**) analyses showed that TGF-β2 levels were significantly decreased in FMOD-null granulation tissue at day 7 post-injury. Overall, TGF-β2 expression in FMOD-null wounds was higher at the early wound edge but lower after wound closure compared with that in WT wounds.

### TGF-β3 expression in FMOD-null mice is boosted at day 0.5 post-injury but becomes lower than WT expression at day 14 post-injury

In WT and FMOD-null mice, inflammatory cell TGF-β3 staining was negligible in unwounded tissue, increased during days 1 and 2 post-injury, and returned to the baseline levels after wound closure (**Table 3**). Similar to TGF-β1 and -β2, unwounded skin showed strong TGF-β3 staining in epidermis and adjacent hair follicles of both WT and FMOD-null groups (**Table 3**), but the TGF-β3 expression dwindled by day 0.5 post-injury (**Table 3**). As described in our previous observation [14], in contrast to WT wounds with negligible ECM and fibroblast TGF-β3 staining, FMOD-null wounds displayed strong TGF-β3 signals in both the ECM and ∼25% of dermal fibroblasts at day 0.5 post-injury (**Figure 2C** and **Table 3**). This finding correlated with higher total dermal TGF-β3 protein expression (**Figure 3E**) and mRNA transcription at day 0.5 (**Figure 3F**). At day 14 post-injury, WT fibroblasts displayed strong TGF-β3 staining while FMOD-null fibroblasts only exhibited negligible signals (**Figure 2C** and **Table 3**). Thus, TGF-β3 expression was relatively higher in WT wounds than FMOD-null wounds at day 14 post-injury (**Figure 3E, F**).

### Expression of TGF-β receptors is increased in FMOD-null wounds at early stage of wound healing

For WT and FMOD-null mice, individual inflammatory cell TβRI [*aka.* activin receptor-like kinase 5, (ALK5)] staining was negligible in unwounded tissues. At day 0.5 post-injury, inflammatory cells exhibited moderate TβRI staining in FMOD-null wounds but negligible staining in WT wounds (**Table 4**). From days 1 to 2 post-injury, inflammatory cells of both groups presented strong TβRI signals, which returned to negligible levels by day 7 (**Table 4**). Meanwhile, strong TβRI staining was found in the migrating epidermis of FMOD-null mice at day 1 post-injury but not in WT controls (**Figure 2D** and **Table 4**). Interestingly, elevated expression of lumican – another class II SLRP that binds to TβRI and thus promotes epithelial migration [48] – was also found in the FMOD-null migrating epidermis as evidenced by IHC staining (**Figure S1**). As we reported previously [14], more dermal fibroblasts strongly expressing TβRI were found in FMOD-null wounds than in WT wounds, although similar staining density was observed in both wounds (**Figure 2D** and **Table 4**). Unwounded WT and FMOD-null skin tissues had similar total TβRI expression (**Figure 4A, B**). After injury, *TβRI* transcripts of WT mice peaked at day 0.5 post-injury and slowly decreased to baseline levels by day 14 (**Figure 2B**). *TβRI* transcripts of FMOD-null mice also peaked at 0.5 day post-injury, but were ∼1.84 times WT *TβRI* transcripts at the same time point (**Figure 4B**). FMOD-null *TβRI* mRNA levels then rapidly declined to baseline by day 1, but rebounded at days 7 and 14 (**Figure 4B**). Generally, the trends in TβRI protein expression paralleled its mRNA transcription pattern, but were relatively delayed and less dramatic in the degree of changes. Overall, total dermal cell TβRI protein was significantly increased in FMOD-null wounds relative to WT wounds at 0.5, 1, 2 and 14 days after injury (**Figure 4A**).

WT and FMOD-null wounds exhibited similar TβRII staining intensities for individual inflammatory cells at all examined time points (**Table 4**). Our previous studies [14] have already demonstrated that TβRII staining in unwounded WT epidermis, hair follicles, and dermal fibroblasts transiently drops to negligible levels at days 0.5 and 1 post-injury before returning to strong levels by day 2 (**Figure 2E** and **Table 4**). On the contrary, the strong TβRII staining in unwounded FMOD-null epidermis and hair follicles persisted at the same levels during the entire 14-day experimental period (**Figure 2E** and **Table 4**). In addition, FMOD-null fibroblasts exhibited strong TβRII signals from days 0.5 to 7 post-injury until receding to minimal levels of unwounded skin at day 14 (**Figure 2E** and **Table 4**). Similar to TβRI (**Figure 4A, B**), there was a lag phase between the peak mRNA transcription and protein expression of TβRII (**Figure 4C, D**). The reason behind the lag between TβRII mRNA and protein expression is unclear, but it has been described in a mouse embryonic model [49]. In sum, these data demonstrated that FMOD-null wounds have higher TβRII expression than WT wounds during the first 24 hours post-injury.

The epidermis and hair follicles of unwounded WT and FMOD-null mice exhibited strong type III TGF-β receptor (TβRIII, *aka.* betaglycan) staining (**Table 4**). Both genotypes lost TβRIII signals in the migrating epidermis at day 0.5 post-injury but re-gained strong baseline levels by day 1 for FMOD-null wounds and day 2 for WT wounds (**Figure 2F** and **Table 4**). WT hair follicles demonstrated minimal (day 1) to strong (day 14) TβRIII staining, while strong TβRIII signals were observed in FMOD-null hair follicles from days 1 to 14 (**Table 4**). No obvious TβRIII staining was detected in dermal inflammatory cells, ECM, or fibroblasts in either genotype during the entire experimental period (**Table 4**). As such, no significant deviation from the baseline for total TβRIII expression was detected (**Figure 4E, F**). Meanwhile, the lack of ECM staining indicated that the majority of TβRIII protein existed in the membrane-anchored form rather than the soluble form [50]. Taken together, all three TβRs were upregulated in FMOD-null wounds in comparison with WT wounds, especially at early wound healing stages.

### TβRI is essential in TGF-β modulation of dermal fibroblast migration

**Figure 5:**
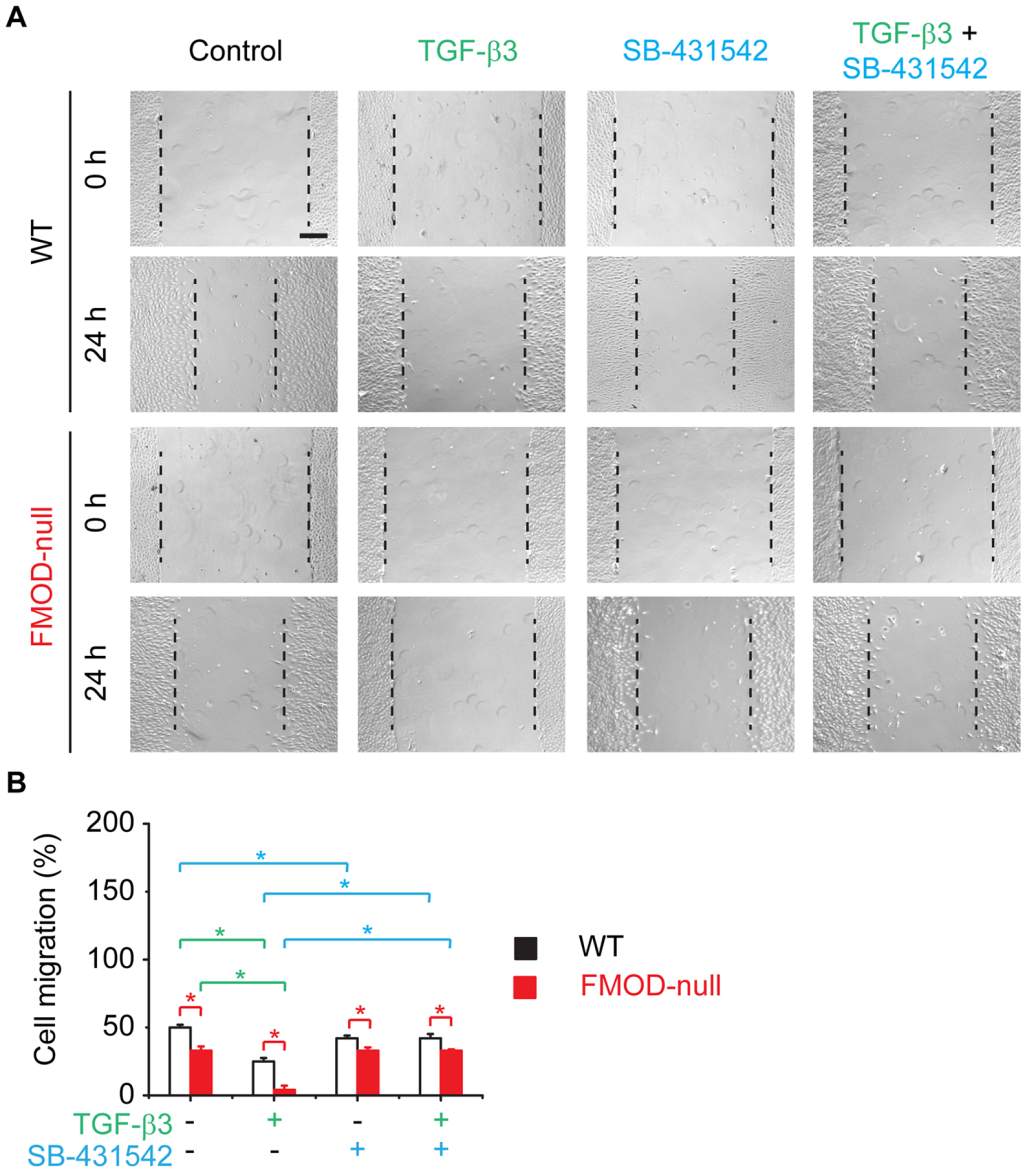
*In vitro* migration assay of primary dermal fibroblasts derived from adult WT and FMOD-null mice skin. Cell migration was documented by photographs taken immediately after scraping, as well as 24(**A**). Migration was quantified by measuring the average wound gap between the wound edges before and after the treatment, and calculated as: Cell migration (%)  =  (Gap_0h_-Gap_24h_)/Gap_0h_ ×100% (**B**). 100 pM TGF-β3 was used to inhibit dermal fibroblast migration *in vitro*, while 10 µM TβRI-specific inhibitor SB-431542 was used to block TβRI-mediated signal transduction. Bar  = 200 µm. N = 6; *, *P*<0.05. Red stars indicate the significance that resulted from FMOD-deficiency; green stars indicate the significance that resulted from TGF-β3 application; and blue stars indicate the significance that resulted from SA-431542 blockage of TβRI.

Consistent with our previous studies [14], FMOD-null dermal fibroblasts demonstrated significantly less motility in comparison with WT controls (**Figure S2** and **Figure 5**). TGF-β1 restored FMOD-null fibroblast migration to PBS-treated WT levels, while TGF-β1 markedly increased WT dermal fibroblast migration (**Figure S2**). Although TβRI-specific inhibitor SB-431542 reduced WT fibroblast migration, SB-431542 did not considerably influence FMOD-null dermal fibroblast migration (**Figure S2**). However, SB-431542 markedly attenuated the pro-migratory effects of TGF-β1 on both WT and FMOD-null fibroblasts (**Figure S2**). On the other hand, TGF-β3 significantly inhibited WT and FMOD-null dermal fibroblast migration to 50% and 8%, respectively, of PBS control levels (**Figure 5**). Surprisingly, SB-431542 entirely prevented TGF-β3's inhibition on WT and FMOD-null fibroblast migration (**Figure 5**), indicating that the anti-motility effect of TGF-β3 on dermal fibroblasts is dependent on TβRI-mediated signal transduction.

## Discussion

In a rat fetal wound model, we previously demonstrated elevated FMOD expression as a potential mechanism for scarless healing in fetal wounds at day 16 of gestation (E16) relative to scarring E19 wounds [41]. Moreover, we restored scarless repair in normally scarring E19 skin wounds by FMOD protein application, while FMOD blockade by anti-FMOD antibodies induced scar in normally scarless E16 wounds [40]. With respect to adult wound repair, the role of FMOD in regulating collagen fibrillogenesis was elucidated in our previous studies using FMOD-null mice, which exhibited loose package collagen fiber networks with increased fibril diameter [42]. We also found that FMOD-deficiency dramatically altered adult mouse wound healing, leading to delayed dermal cell migration, granulation tissue formation and wound closure, decreased vascularity, and increased scar size accompanied by less orderly collagen architecture [14], [42]. Based on these findings, adenoviral FMOD administration was used to ameliorate adult rabbit wound healing [51]. In addition, recent studies revealed that FMOD expression is reduced in human post-burn hypertrophic scar [52], [53]. These studies indicate that FMOD is profoundly involved in cutaneous wound healing.

Remarkably, FMOD and TGF-β1 levels showed inverse trends in both fetal and adult wound healing processes (as one increases the other decreases and *vice versa*) [40], [41], [43], [53]. Specifically, in adult wounded mice, higher TβRI levels in FMOD-null inflammatory cells indicate that FMOD-deficiency enhanced chemotactic response of these cells towards TGF-β ligands [54], [55], which may have contributed to the successively increased inflammatory cell recruitment in FMOD-null animals soon after injury. In addition, TGF-βs could have promoted inflammatory infiltration either directly or by inducing inflammatory cell activation and synthesis of multifunctional cytokines (such as interleukins, tumor necrosis factor-α, and platelet-derived growth factor) [54]–[56]. Since inflammatory cells that initially infiltrated FMOD-null wounds exhibited higher sensitivity to TGF-βs than those in WT wounds due to the increased TβRI expression, the inflammatory autocrine network may have been strengthened [54], [55], [57], resulting in the relatively exaggerated inflammatory response in FMOD-deficient animals during the entire wound healing period. It is worthy of note that the infiltrating inflammatory cells not only combat invading pathogens but also participate in tissue degradation and reestablishment [58]. Therefore, alteration of inflammatory infiltration may have profound influence on downstream migration, proliferation, differentiation, and ultimately the quality of the healing response. For instance, inflammatory cytokines induced by TGF-βs during the inflammatory stage initialize epithelialization in cutaneous wound healing [5], which may be one reason for faster epithelial migration in FMOD-null wounds relative to WT controls [14]. Because TGF-β1 stimulates epidermal cell migration [59]–[62], earlier and increased expression of TGF-β1, TβRI, and TβRII in migrating epidermal tongue may have promoted FMOD-null epithelial migration. Meanwhile, although TβRIII itself has no inherent signaling function, membrane-anchored TβRIII enhances TGF-β signal transduction by presenting TGF-β ligands to TβRII [63]. Thus, expression of membrane-anchored TβRIII in FMOD-null migrating epidermal tongue may also have contributed to the increased epithelial migration. Although FMOD and lumican share 47% amino acid sequence identity and the same collagen-binding region [64], they have inverse expression trends in connective tissues and different functions in the regulation of ECM assembly and cellular behavior [36], [65]–[68]. For instance, we observed increased lumican expression in FMOD-null wound migrating epidermis in our current study. Since lumican promotes epithelial migration *via* binding to TβRI [48], the co-localization and co-elevation of lumican and TβRI could be an additional reason for the accelerated epidermal migration of FMOD-null wounds.

Once epidermal cells begin migrating, epidermal and dermal cells no longer remain adhered to one another, and this disconnection allows the lateral movement of epidermal cells over the wound matrix [3], [7], [69]. Importantly, during the epithelialization period, ECM proteins such as fibronectin together with type I collagen provide the ‘railroad tracks’ on top of dermis that facilitate migrating epidermal cells to separate desiccated eschar from viable tissue and reestablish the epithelial layer [3], [7], [69]. However, our previous studies have shown that expression of both fibronectin and type I collagen is decreased in FMOD-null wounds compared with that in WT wounds before wound closure [14]. Instead of migrating on the ECM layer deposited by dermal cells that have newly moved into the wound area as in WT wounds, epidermal cells migrated more intimately on dermis in FMOD-null wounds, hindering normal establishment of viable epithelial layer into the wounds [14]. Moreover, TGF-β3 selectively halts dermal fibroblast proliferation and migration and leads to retarded dermal cell entry [12]–[14]. In our current study, we have shown that TβRI plays an essential role in TGF-β3-mediated inhibition of FMOD-null dermal fibroblast migration. Additionally, Bandyopadhyay *et al.* demonstrated that the level of TβRII determines the uptake of the anti-motility signal of TGF-β3 on dermal fibroblasts [12]. Hence, high TGF-β3, TβRI, and TβRII levels in FMOD-null dermis resulted in lack of accompanying dermal cell migration and proliferation and subsequently delayed granulation tissue formation, leading to a greater wound surface area requiring epithelialization [12]–[14]. Taken together, despite that epithelial migration was accelerated in FMOD-null wounds, the need for migration through a large, deep, U-shaped dermal concavity extending from superficial dermis to subcutaneous fat delayed epithelialization and led to an excessively exuberant fibroproliferative response after wound closure to cause extensive scarring [14].

**Figure 6:**
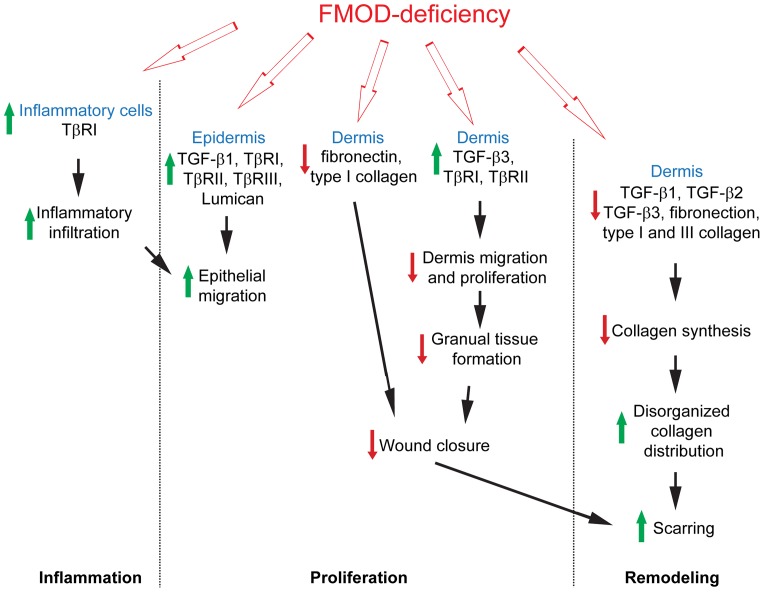
A brief summary of the major effects of FMOD-deficiency on adult mouse wound healing.

Furthermore, the production capability of all three TGF-β ligands by individual dermal cells was reduced during the remodeling stage in FMOD-null wounds accompanied by a decrease in collagen synthesis, which is predominantly stimulated by TGF-β [14]. Adding to the increased fibrotic cell density, the abnormal TGF-β expression may have further contributed to the disorganized collagen distribution in FMOD-null scars [42]. Meanwhile, previous studies have shown that FMOD activates the classical and the alternative pathways of complement [32], [70]. Considering that administration of complement components in acute injury models promotes wound healing by enhancing angiogenesis and wound strength [71], [72], it is possible that the weakened activation of the complement cascade caused by FMOD-deficiency led to a less vascularized, less strengthened scar in FMOD-null mice. Therefore, the present study clearly provides valuable insight into the novel and complex role of FMOD in orchestrating TGF-β bioactivity during wound repair and reveals that FMOD critically coordinates temporospatial distribution of TGF-β ligands and receptors in various cell types during the entire adult mouse wound healing process (**Figure 6**).

Since its discovery, TGF-β has been shown to have cell-specific effects on cellular proliferation, differentiation, and metabolism [8], [73]–[76]. In particular, TGF-β is known to regulate wound repair and can be produced by a variety of cells that are active in the wound healing process [5], [9], [75], [77]. In addition, the expression level and pattern of TGF-β ligands and receptors are differentially regulated during normal and impaired wound healing [5], [78], [79], which suggests that the concomitant expression of TGF-β isoforms and their signal-transducing receptors govern potential TGF-β activities. In this study, a single missing gene, *FMOD*, leads to marked alterations in wound healing phenotype as well as in temporospatial expression of TGF-β ligands and receptors throughout the wound repair process. The profound alterations in FMOD-null mice demonstrate that TGF-β signaling is contextually dependent on relative ligand, receptor and modulator (e.g., FMOD) ratios, and that the onset, degree, distribution and duration of TGF-βs are crucial to the wound healing process.

As a broadly distributed ECM component, FMOD exerts functions in a variety of biological processes. All SLRP members, including FMOD, have a central protein core made up of 6–10 leucine-rich repeats (LRRs) flanked by cysteine-clusters and substituted with covalently lined glycosaminoglycan (GAG) side chains [32], [33]. They are able to bind to various types of collagens, thereby regulating the kinetics, assembly, and special organization of fibrils and protecting collagen fibrils from cleavage by collagenases [32], [33]. For instance, FMOD-deficient mice have impaired collagen fibrillogenesis in tendon and predentin in addition to dermis [36], [65], [80]. However, the biological functions of SLRPs extend far beyond their interactions with collagens since they also interact with various growth factors and cytokines, allowing modulation of their diverse functions [32]–[34]. For example, in agreement with our previous research [14], [40], [41], this study demonstrates that FMOD interacts with TGF-βs to modulate the cellular responses in cutaneous wound healing. In addition, our recent studies have revealed that FMOD can promote angiogenesis [68], which is essential in wound healing especially during the remodeling stage. FMOD also offers potential therapeutic benefits in saphenous vein graft failure by reducing associated neointima formation [81]. Thus, FMOD is a potential agent that can aid tissue regeneration. Moreover, Oldberg *et al.* found that FMOD determines carcinoma stroma matrix structure and fluid balance in carcinoma [82]. Other studies revealed FMOD as a novel tumor-associated antigen in leukemia, lymphoma, and leiomyoma [83]–[85]. Hence, FMOD may additionally play a vital role in cancer diagnosis and treatment. Finally, FMOD has been found to be integral in maintenance of endogenous stem cell niches [35], and we have discovered that continual treatment with FMOD in a serum-free condition is sufficient to reprogram somatic cells to a minimally proliferative, multipotent stage [38]. Thus, FMOD has an extensive function in cell fate determination as well. On the other hand, upregulation of FMOD in inflamed joint and gingival tissues indicates that FMOD is also involved in sustained inflammation, possibly *via* activating the complement cascade [70], [86]. Taken together, FMOD as well as other SLRP family members are implicated in diverse biological functions during development and in various pathologies. Future research should aim at translating the rapidly expanding knowledge on SLRPs into the clinical setting by elucidating the detailed mechanisms underlying their functions and defining therapeutic strategies for inflammatory, fibrotic, and malignant disorders.

## Supporting Information

Figure S1 **IHC staining of lumican in wounded WT and FMOD-null adult mice skin at 1 day post-injury.** Inserts show low magnification view. Black triangles: migrating epidermal tongues. Bar  = 100 µm.
(TIF)

Figure S2 ***In vitro*** **migration assay of primary dermal fibroblasts derived from adult WT and FMOD-null mice skin.** Cell migration was documented by photographs taken immediately after scraping, as well as 24 h later (**A**). Migration was quantified by measuring the average wound gap between the wound edges before and after the treatment, and calculated as: Cell migration (%)  =  (Gap_0h_-Gap_24h_)/Gap_0h_ ×100% (**B**). 100 pM TGF-β1 and/or 10 µM TβRI-specific inhibitor SB-431542 were used. Bar  = 200 µm. N = 6; *, *P*<0.05. Red stars indicate the significance that resulted from FMOD-deficiency; yellow stars indicate the significance that resulted from TGF-β1 application; and blue stars indicate the significance that resulted from SA-431542 blockage of TβRI.
(TIF)
