# Correction: Fibromodulin-Deficiency Alters Temporospatial Expression Patterns of Transforming Growth Factor-β Ligands and Receptors during Adult Mouse Skin Wound Healing

**DOI:** 10.1371/journal.pone.0246557

**Published:** 2021-01-29

**Authors:** The PLOS ONE Staff

In Fig 2E, the image labelled WT is a duplicate of Fig 2F. The authors have provided a corrected version here. The publisher apologizes for the error.

**Fig 2:**
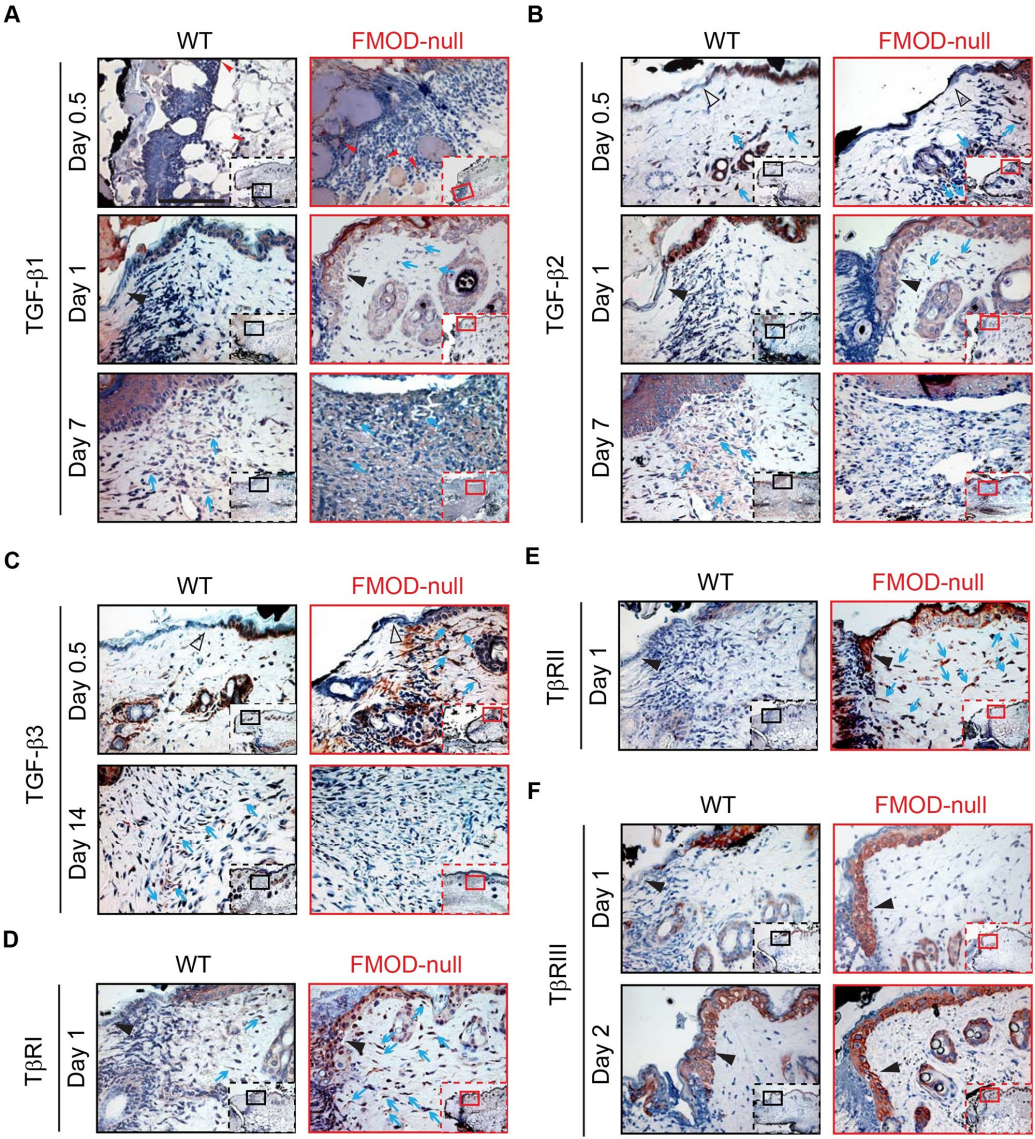
Immunohistochemical (IHC) staining of wounded WT and FMOD-null adult mice skin. (A) TGF-β1, (B) TGF-β2, (C) TGF-β3, (D) TβRI, (E) TβRII, and (F) TβRIII. Inserts show low magnification view. Red arrowheads: inflammatory cells; open black triangles: epidermis at wound edge; solid black triangles: migrating epidermal tongues; blue arrows: dermal fibroblasts. Bar = 100 μm.
