# Supplementary figures and images for: Fibromodulin-Deficiency Alters Temporospatial Expression Patterns of Transforming Growth Factor-β Ligands and Receptors during Adult Mouse Skin Wound Healing
Source: PLoS One. 2014 Mar 6;9(3):e90817. doi: 10.1371/journal.pone.0090817 (PMC3948369; doi:10.1371/journal.pone.0090817)

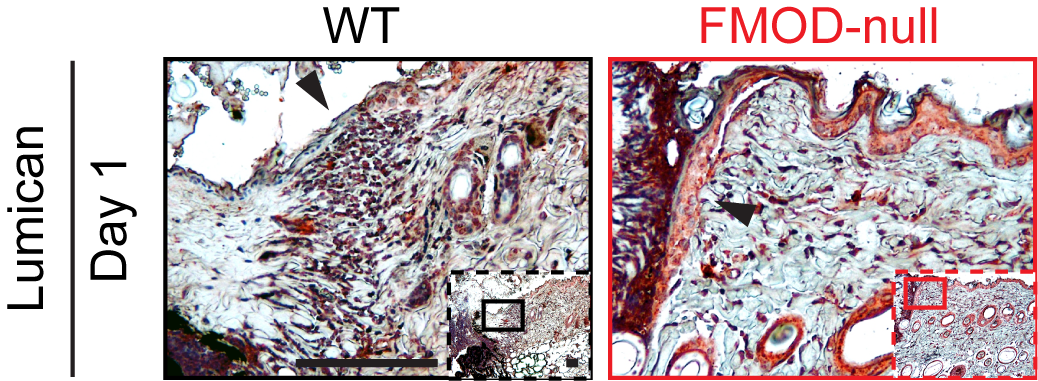

Supplement: Figure S1 — IHC staining of lumican in wounded WT and FMOD-null adult mice skin at 1 day post-injury. Inserts show low magnification view. Black triangles: migrating epidermal tongues. Bar = 100 µm. (TIF) [file pone.0090817.s001.tif]

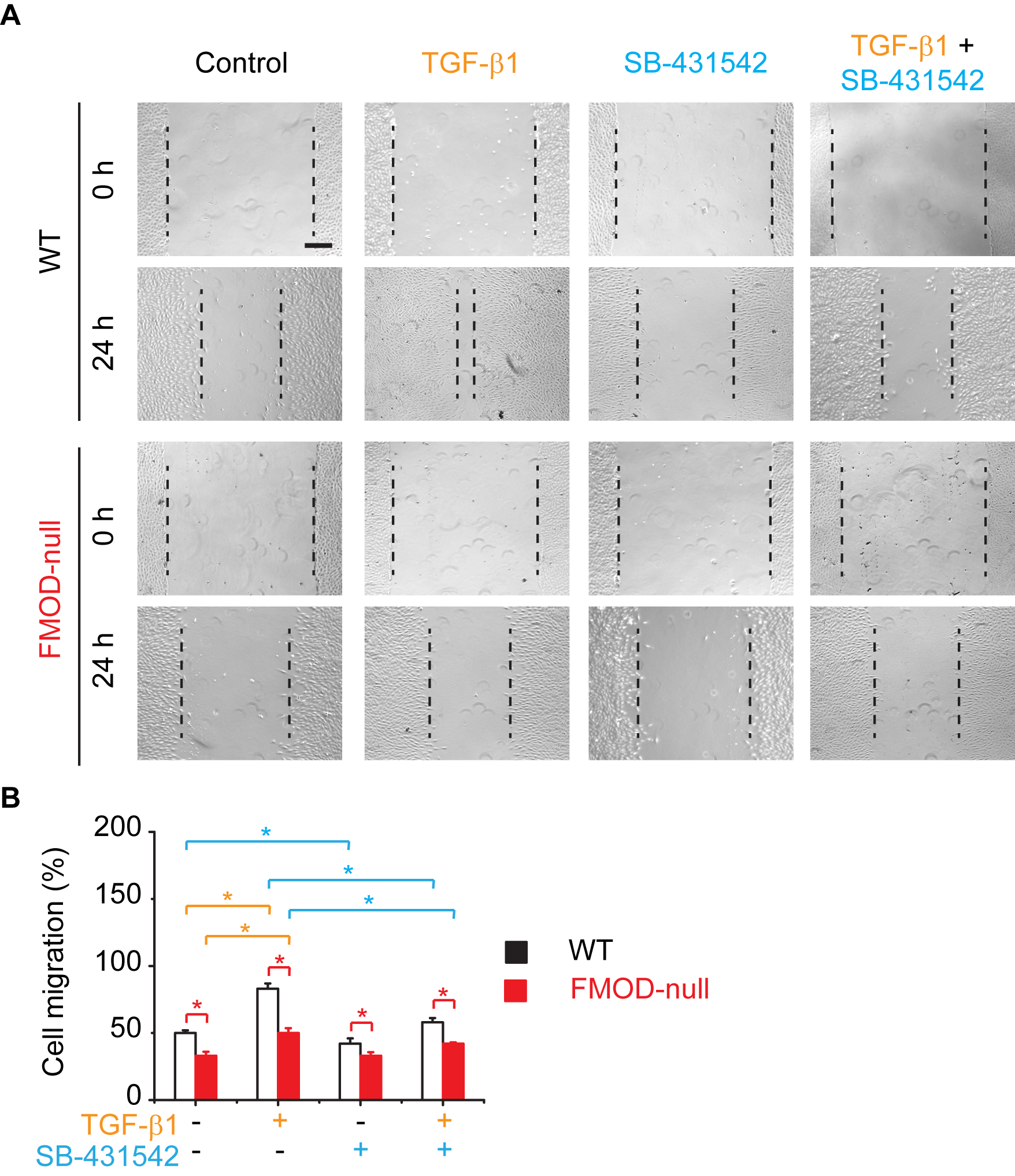

Supplement: Figure S2 — In vitro migration assay of primary dermal fibroblasts derived from adult WT and FMOD-null mice skin. Cell migration was documented by photographs taken immediately after scraping, as well as 24 h later (A). Migration was quantified by measuring the average wound gap between the wound edges before and after the treatment, and calculated as: Cell migration (%) = (Gap0h-Gap24h)/Gap0h ×100% (B). 100 pM TGF-β1 and/or 10 µM TβRI-specific inhibitor SB-431542 were used. Bar = 200 µm. N = 6; *, P<0.05. Red stars indicate the significance that resulted from FMOD-deficiency; yellow stars indicate the significance that resulted from TGF-β1 application; and blue stars indicate the significance that resulted from SA-431542 blockage of TβRI. (TIF) [file pone.0090817.s002.tif]
